# Diffractive multifocal intraocular lens implantation in eyes with a small-diameter pupil

**DOI:** 10.1038/s41598-018-30141-1

**Published:** 2018-08-03

**Authors:** Masayuki Ouchi, Takuya Shiba

**Affiliations:** 1 Ouchi Eye Clinic, Kyoto, Japan; 2 Department of Ophthalmology, Kyoto Prefectural University of Medicine, Kyoto, Japan; 3 Department of Ophthalmology, The Jikei university school of medicine, Tokyo, Japan

## Abstract

Postoperative outcome of diffractive multifocal intraocular lens (MIOL) implantation in eyes with a small-diameter pupil was evaluated. This non randomized case series involved 23 eyes of 23 patients who underwent diffractive MIOL implantation and whose preoperative photopic pupil diameter was ≤3.0 mm [small-diameter pupil (SDP) group], and 79 eyes of 79 patients implanted with the same MIOL whose pupil diameter was >3.0 mm as controls (LDP group). Contrast sensitivity of high spatial frequency (12, and 18 cycle per degree) and both 12.5% and 6% low-contrast visual acuity (VA) were significantly worse in the SDP group than in the LDP group (*P* = 0.04, 0.05, 0.05, 0.03). However, no significant difference was found between eyes in the SDP group with a postoperative pupil diameter greater than 3.0 mm and LDP group eyes. No significant differences were found in uncorrected and corrected distance VA (UDVA, CDVA), uncorrected and corrected near VA (UNVA, CNVA), contrast sensitivity with low spatial frequency, and defocus curve between the two groups. In conclusion, in eyes implanted with a diffractive MIOL, a pupil diameter of ≤3.0 mm deteriorates contrast sensitivity. This effect was eliminated postoperatively when pupil size was enlarged to >3.0 mm during surgery.

## Introduction

Multifocal intraocular lens (MIOL) implantation is now widely applied for the treatment of cataracts. The indications for the use of MIOLs, however, must be more carefully assessed compared with those for monofocal IOLs. In patients who undergo MIOL implantation, preoperative astigmatism, patient age and occupation, eye diseases other than cataracts as well as dry eye, and pupil size should be considered.

Clinical studies report that in patients with a small pupil, refractive MIOL implantation may result in poor postoperative near-field visual function^1–3^. Fujimoto *et al*.^4^ reported a case series of laser pupilloplasty for eyes with decreased near visual acuity (VA) due to age-related narrowing of the pupil in patients implanted with a refractive MIOL. A preoperative photopic pupil greater than 3.0 mm in size is generally considered a required indication for refractive MIOL implantation. On the other hand, diffractive MIOLs are grouped into mainly two categories: (1) apodized diffractive-refractive IOLs: allocate diffractive zone only in central area with non-constant step height from the center to the periphery: and (2) full-optic diffractive IOLs: diffractive zone is applied to the entire optical surface with constant step height:. Artigas *et al*.^5^ reported that modulation transfer function measurements revealed deterioration with a small pupil, using diffractive IOL implanted optical bench. Alfonso *et al*.^6^ reported that pupil size is related to contrast sensitivity in eyes implanted with an apodized diffractive-refractive IOL, but to the best of our knowledge there are no published clinical case series studies of eyes with a small pupil implanted with a full-optic diffractive IOL.

In the present study, we evaluated the postoperative outcome of full-optic diffractive MIOL implantation in eyes with a preoperative small pupil, as well as the correlation between postoperative pupil size and visual function.

## Patients and Methods

This retrospective study was carried out at a single institute, and ethics approval was obtained from the Institutional Review Board of Ouchi Eye Clinic. Prior written informed consent for the use of medical records was obtained from all patients, and patient data were used in accordance with the tenets of the Declaration of Helsinki.

The medical records of consecutive patients who underwent cataract surgery and diffractive MIOL TECNIS^®^ ZMB00 (Abbott Medical Optics, Santa Ana, CA) with 6.0 mm optical zone with bifocal 4.0 diopter (D) near add power implantation from January 2014 through December 2016 were reviewed. Eyes with a best-corrected distance VA (CDVA) greater than 0.1 logarithm of the minimum angle of resolution (Log MAR) were excluded from the study.

This study involved 23 eyes of 23 patients with a preoperative photopic pupil size of 3.0 mm or less (small-diameter pupil [SDP] group) and 79 eyes of 79 patients with a preoperative pupil size of greater than 3.0 mm (large-diameter pupil [LDP] group, controls) who underwent ZMB00 MIOL implantation and complete postoperative follow-up measurements. We randomly selected one eye for statistical analysis in bilaterally implanted patients. The postoperative outcomes of the SDP-group eyes and the LDP-group eyes were then compared.

### Surgical Procedures

All surgeries were performed by a single surgeon (M.O.) using the same surgical procedure. First, a 2.2-mm clear corneal incision was made with a stainless steel keratome, followed by a continuous curvilinear capsulorhexis (approximately 5.6 mm in diameter) using capsulorhexis forceps. After thorough hydro dissection, endocapsular phacoemulsification of the nucleus and aspiration of the cortex were performed. The lens capsule was inflated with 1% sodium hyaluronate (HEALON^®^, Abbott Laboratories Inc., Abbott Park, IL), and the IOL was then implanted in the capsular bag using an INTREPID^®^ AutoSert^®^ IOL Injector (Alcon Laboratories, Inc., Fort Worth, TX) automated system. In eyes with pre-operative pupil of less than 2.3 mm in size, multiple small sphincterotomies were performed after IOL implantation. In cases requiring multiple small sphincterotomies, after injecting the viscoelastic agent (HEALON^®^), micro capsule scissors were inserted via the main 2.2-mm incision, a side port at 2-o’clock, and a newly created port at 6-o’clock for sphincterotomy beneath the main incision, and 20 to 25 tiny sphincterotomies were cut at equal intervals of 15° around the pupillary border.

### Outcome Measures

Prior to surgery, all patients underwent ophthalmologic examinations including uncorrected distance VA (UDVA) and CDVA at 5.0 m, refractive sphere, non-contact tonometry, slit-lamp evaluation, fundoscopy, corneal topography, biometry (IOL Master, version 4.3; Carl Zeiss Meditec AG, Jena, Germany), and pupil size. All patients were then evaluated postoperatively at 3 months with the same examinations performed preoperatively, as well as uncorrected and corrected near VA, defocus curve, contrast sensitivity, and low contrast VA with 12.5% and 6% contrast.

The objective refractive status (spherical and cylindrical powers) and keratometric cylinder were measured using an auto refractometer (ARK-560A; NIDEK Co., Ltd., Gamagori, Japan). The manifest spherical equivalent was determined as the spherical power plus one-half the cylindrical power. Pupil size was measured using an ARK-10000 (NIDEK), whose accuracy for pupil size measurement is comparable to that of the Procyon pupilometer^7^. Pupil size was measured three times under the condition that ambient room illumination was <1lux determined using the Minolta Chroma meter (Minolta, Glen Cove, NY) and the luminance emitted by the ARK-10000 was 100 to 150 cd/m^2^, and the mean value was calculated.

Contrast sensitivity testing was performed using a CSV-1000 (VectorVision, Greenville, OH) contrast sensitivity instrument under mesopic conditions with chart lighting of 2 cd/mm^2^. Low-contrast VA was measured with System Chart (SC-1600; NIDEK), and the defocus curve was obtained at a distance of 5 m with monocular vision and the patient’s distance correction. Spherical lenses were added in 0.5D steps from +2.0D to −6.0D, and VA was recorded for each type of blur. All recorded information was represented in a two-dimensional graph.

### Statistical Analysis

Statistical analyses were performed using R version3.4.1 (The R Foundation: https://www.r-project.org/). When the values of each dataset followed a normal distribution, Student’s t-test or Welch’s t-test, depending on the homogeneity of variance, was used for statistical analysis. When the data did not follow a normal distribution, the Mann-Whitney U test was used. Using Spearman correlation analysis, low-contrast VA and contrast sensitivity correlated with the postoperative pupillary diameter. Also Spearman correlation analysis was used to investigate the correlation between pre- and post-operative pupillary diameter. Any differences with a *P*-value less than 0.05 were considered statistically significant.

### Data availability

The datasets generated during and/or analyzed during the current study are available from the corresponding author on reasonable request.

## Results

In all patients, surgeries were performed without complication and all IOLs were implanted in the capsular bag. None of the cases required sutures. Multiple small sphincterotomies were performed in 4 eyes in the SDP group.

The baseline demographic data of the 23 eyes in the SDP group and the 79 eyes in the LDP group are shown in Table 1. The mean preoperative photopic pupil diameter in the SDP group was 2.61 ± 0.25 mm (range: 2.13–2.84 mm) and that of the LDP group was 4.07 ± 0.87 mm (range: 3.01–5.67 mm). The mean postoperative pupil diameter was 2.98 ± 1.22 mm (2.04–3.81 mm) in the SDP group, while 10 of 23 eyes measured larger than 3.00 mm, and 4.01 ± 1.56 mm (3.23–5.04 mm) in the LDP group. LDP group registered significantly larger pupil diameter both preoperatively and postoperatively.

**Table 1:** Baseline data.

	SDP Group	LDP Group	*P*-Value
Mean Age (years) [range]	66.8 ± 11.0 [44–79]	64.9 ± 10.0 [39–80]	0.05
UDVA (log MAR)	0.66 ± 0.41	0.52 ± 0.57	0.42
CDVA (log MAR)	0.13 ± 0.31	0.21 ± 0.30	0.10
Sphere (D)	−2.41 ± 6.12	−2.48 ± 5.81	0.93
Corneal K (D)	0.90 ± 071	0.73 ± 0.54	0.32
Pre-op Pupil size (mm)	2.61 ± 0.25 [2.13–2.84]	4.07 ± 0.87 [3.01–5.67]	<0.001
Post-op Pupil size (mm)	2.98 ± 1.22 [2.04–3.81]	4.01 ± 1.56 [3.23–5.04]	<0.001

SDP = small diameter pupil; LDP = larger diameter pupil; UDVA = uncorrected distance visual acuity (VA); CDVA = best corrected distance VA; Sphere = refractive spherical equivalent; D = diopters; Corneal K = corneal astigmatism.

Values are presented as mean ± SD. Pre-op; preoperative, Post-op; postoperative.

There were significant correlation between pre- and post- operative pupil diameter in LDP group (p = 0.0002), though correlation was not significant in SDP group (p = 0.1).

### Visual Acuity

**Figure 1:**
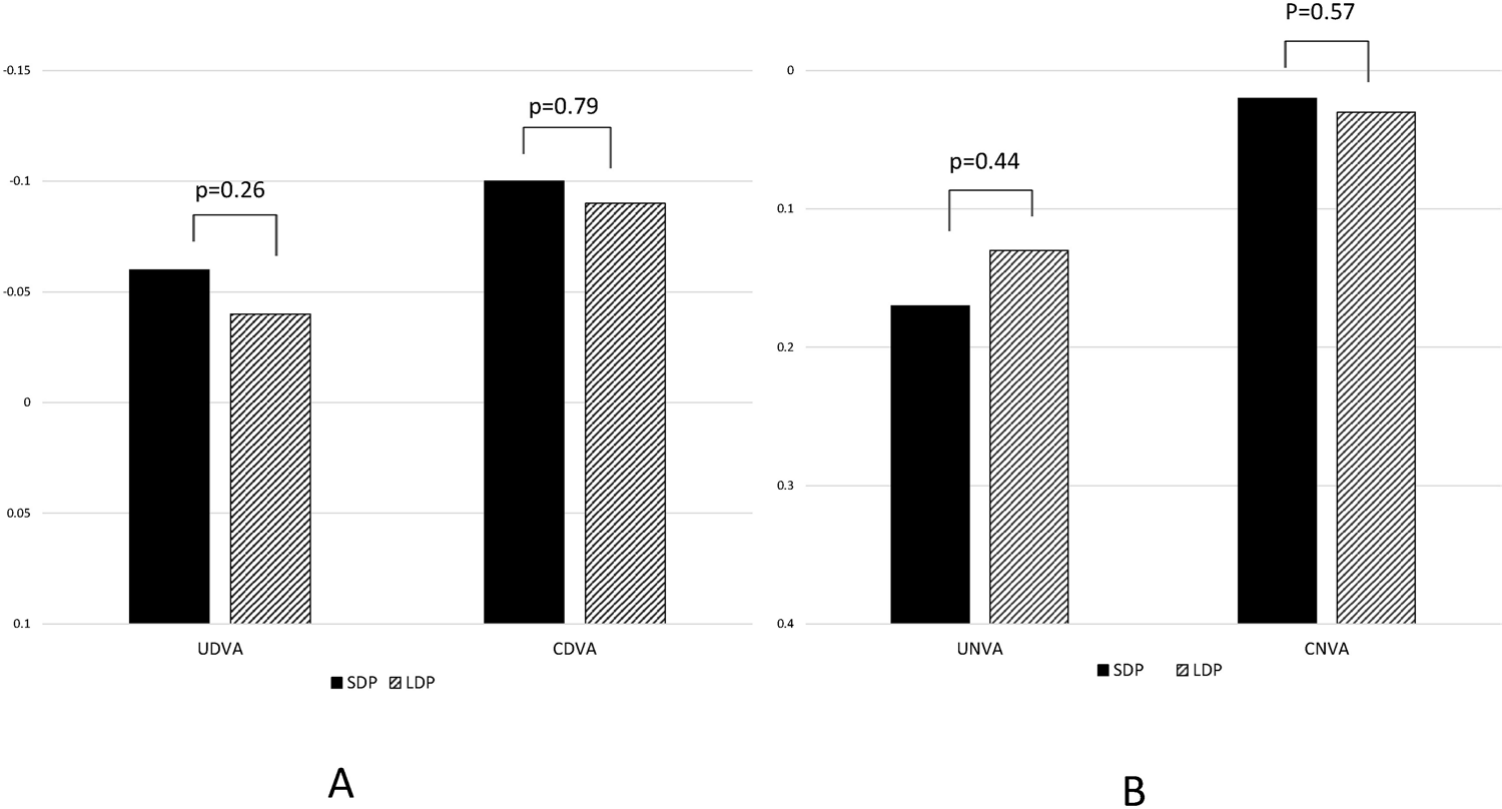
Postoperative visual acuity (VA). No significant difference was observed in UDVA, CDVA, UNVA, and CNVA between the two groups. SDP: eyes with a preoperative pupil size of <3.0 mm. LDP (controls): eyes with a preoperative pupil size ≥3.0 mm. UDVA: uncorrected distance VA, CDVA: best-corrected distance VA, UNVA: uncorrected near VA, CNVA: best-corrected near VA.

**Figure 2:**
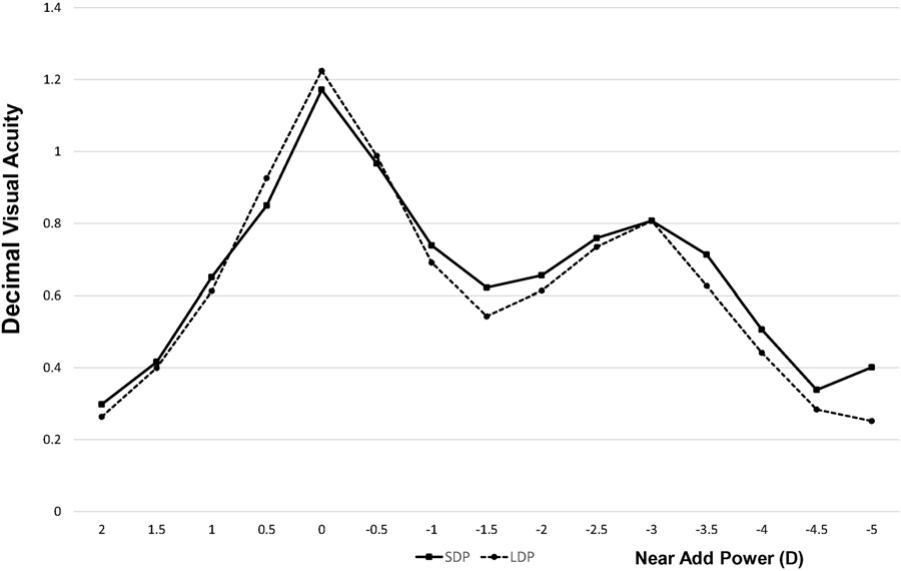
Defocus curve. No significant difference was detected between the two groups in all near additional power. Decimal visual acuity was converted from the mean log MAR VA.

Mean UDVA was −0.06 ± 0.14 in the SDP group and −0.04 ± 0.15 in the LDP group (*P* = 0.27). Mean CDVA was −0.10 ± 0.10 in the SDP group and −0.09 ± 0.11 in the LDP group (*P* = 0.79). Neither UDVA nor CDVA differed significantly between the two groups. The mean uncorrected near VA (UNVA) was 0.17 ± 0.17 in the SDP group and 0.13 ± 0.30 in the LDP group (*P* = 0.44), and best corrected near VA (CNVA) was 0.02 ± 0.11 in the SDP group and 0.03 ± 0.14 in the LDP group (*P* = 0.57; Fig. 1). The defocus curves were similar between the SDP and LDP groups, and no significant difference in the log MAR VA was detected between the two groups in any near additional power (Fig. 2).

### Contrast Sensitivity

**Figure 3:**
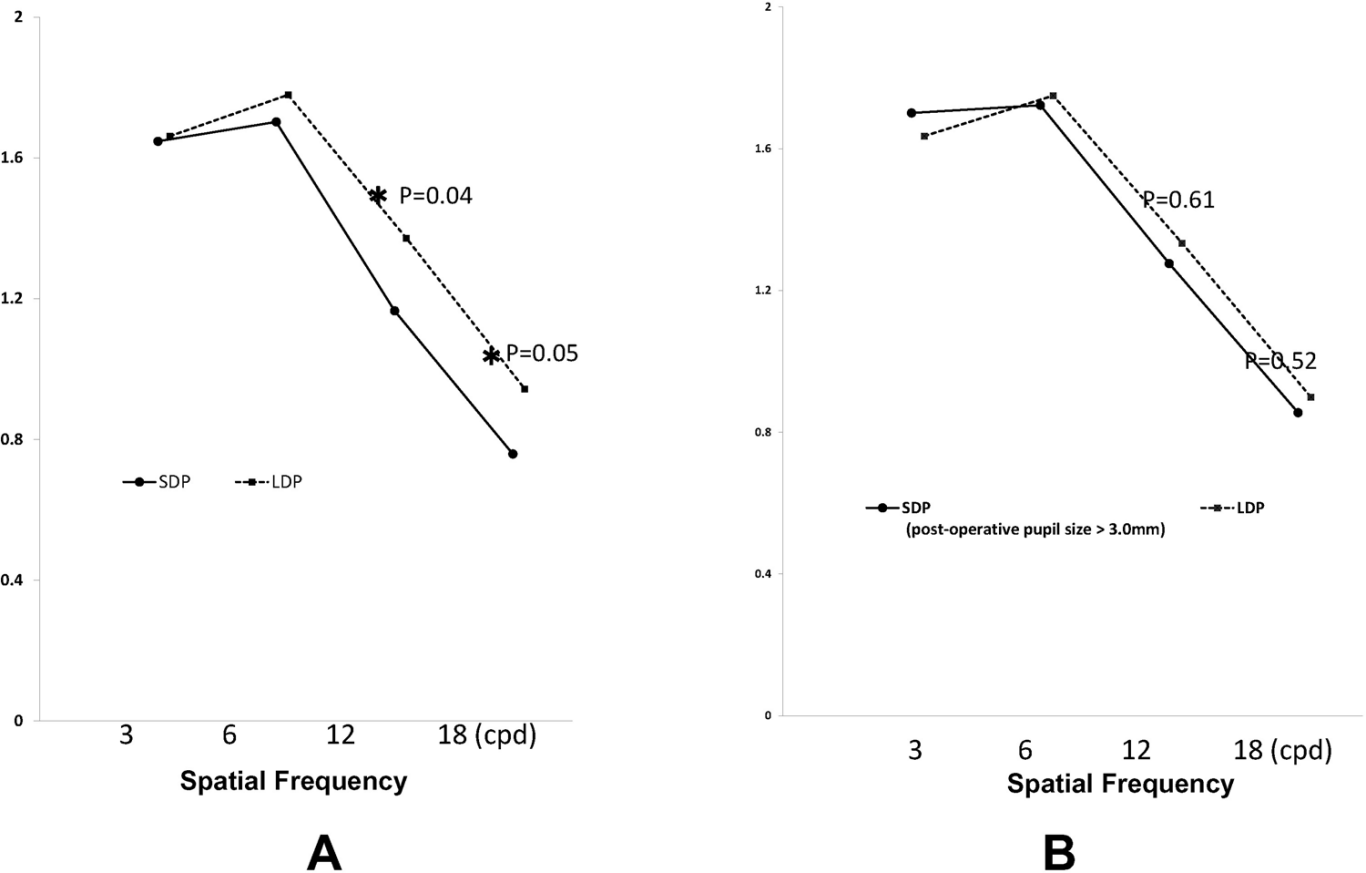
Contrast sensitivity. SDP group eyes registered significantly worse contrast sensitivity at 12 and 18 cycles per degree (cdp) than LDP group eyes **(A)**, but eyes with a postoperative pupil size >3.0 mm among the SDP group were not significantly different from the LDP group eyes **(B)**.

The mean contrast sensitivity at 12 and 18 cycles per degree (cpd) were 1.17 ± 0.21 and 0.76 ± 0.37 in the SDP group, and 1.37 ± 0.31 and 0.94 ± 0.30 in the LDP group. Contrast sensitivity at both 12 and 18 cpd was significantly worse in the SDP group than in the LDP group (*P* = 0.04 and 0.05, respectively: Fig. 3A). Contrast sensitivity at 12 and 18 cpd of all 102 eyes within both groups was distributed with standard deviations of 0.32 and 0.32, respectively. The difference in the means between the two groups was 0.21 and 0.16 in 12 and 18 cpd, respectively, allowing us to reject the null hypothesis that the means of the SDP and LDP are equal with a probability (power) of 1.0 and 0.993, respectively, when the Type I error probability associated with this test is 0.05. However, in comparison between eyes in the SDP group with a postoperative pupil diameter greater than 3.0 mm, and in LDP group eyes, no significant difference in the contrast sensitivity was detected at any spatial frequency (Fig. 3B).

Using Spearman’s correlation coefficient analysis, significant positive correlations were detected between the preoperative pupil diameter and contrast sensitivity at 12 and 18 cpd (Spearman *r* = 0.452, *P* = 0.02, Spearman r = 0.436, *P* = 0.04, respectively) (Table 2).

**Table 2:** Correlation between preoperative pupil size and low contrast VA or contrast sensitivity (Spearman’s correlation coefficient analysis).

	Correlation Coefficients
12.5% low contrast VA	0.407 (*p* = 0.04)
6.0% low contrast VA	0.440 (*p* = 0.03)
Contrast sensitivity (3 cpd)	0.26 (*p* = 0.19)
Contrast sensitivity (6 cpd)	0.15 (*p* = 0.63)
Contrast sensitivity (12 cpd)	0.452 (*p* = 0.02)
Contrast sensitivity (18 cpd)	0.436 (*p* = 0.04)

VA: visual acuity, cpd: cycles per degree.

### Low Contrast (12.5%, 6%) Visual Acuity

**Figure 4:**
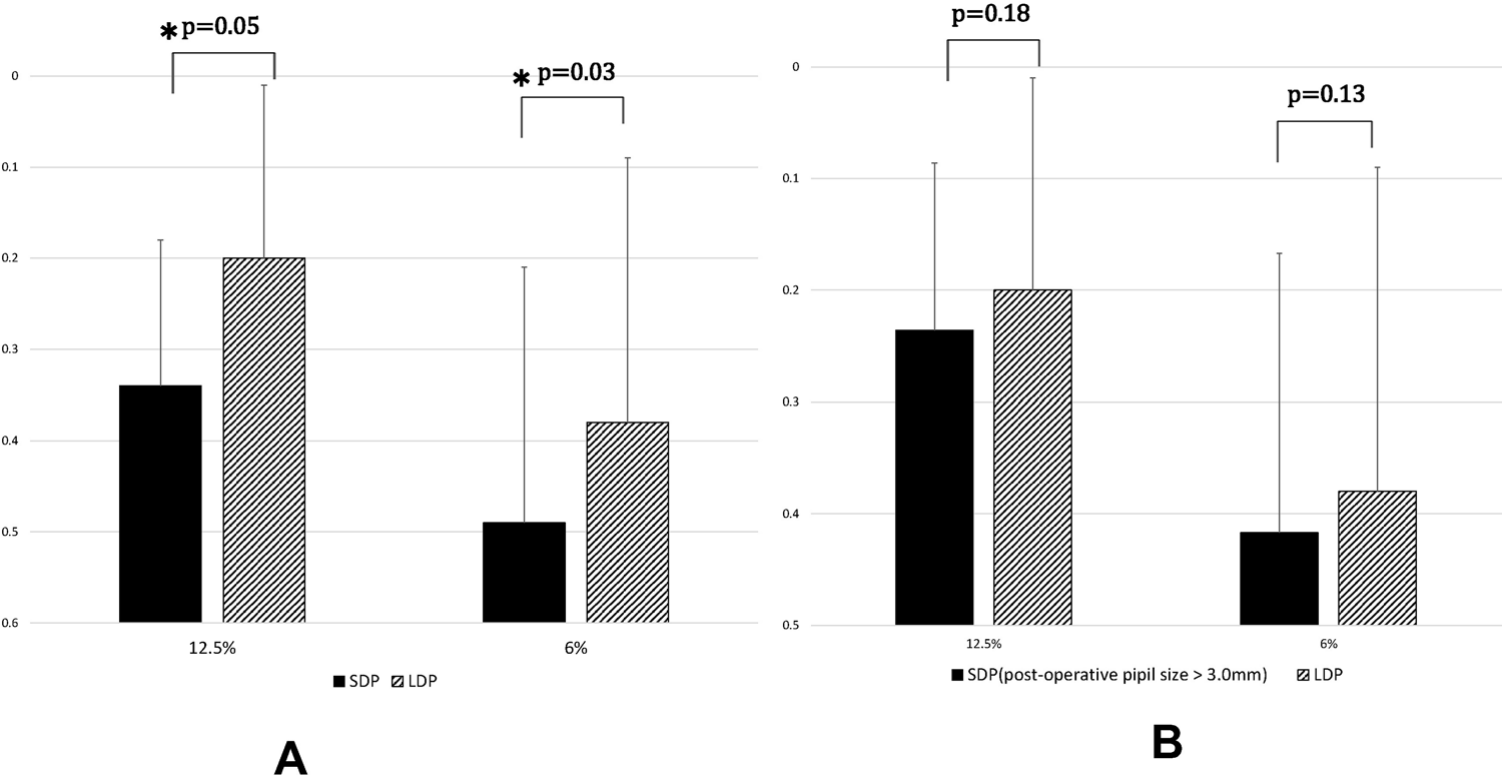
Low contrast VA. 12.5%: VA with 12.5% contrast. 6%: VA with 6% contrast. SDP group eyes registered significantly lower VA than LDP eyes both at 12.5% and 6.0% contrast **(A)**, but the difference was no longer significant when eyes having a postoperative pupil size >3.0 mm among the SDP group were compared to the LDP group eyes **(B)**.

The mean 12.5% low contrast VA was 0.34 ± 0.16 in the SDP group and 0.20 ± 0.19 in the LDP group, and mean 6% low contrast VA was 0.49 ± 0.28 in the SDP group and 0.38 ± 0.29 in the LDP group. Both 12.5% and 6% low contrast VA were significantly worse in the SDP group than in the LDP group (*P* = 0.05, 0.03; Fig. 4A). Low contrast VAs of 12.5% and 6% of all 102 eyes within both groups were distributed with standard deviations of 0.17 and 0.25, respectively. The difference in the means between the two groups was 0.14 and 0.10 at the 12.5% and 6% contrasts, respectively, allowing us to reject the null hypothesis that the means of the SDP and LDP are equal with a probability (power) of 1.00 and 0.979, respectively, when the Type I error probability associated with this test is 0.05. No significant difference was detected in either the 12.5% and 6% contrast VA, however, between eyes in the SDP group with a postoperative pupil diameter greater than 3.0 mm and LDP group eyes (Fig. 4B).

Significant positive correlations were detected between the preoperative pupil diameter and 12.5% or 6% contrast VA (Spearman *r* = 0.407, *P* = 0.04, Spearman r = 0.440, *P* = 0.03, respectively) (Table 2).

## Discussion

The findings of the present study demonstrated that contrast sensitivity at high spatial frequencies and low contrast VA were worse in eyes with a preoperative small-diameter pupil than in eyes with a large-diameter pupil. In addition, these differences were not seen when eyes that acquired a postoperative pupil size of greater than 3.0 mm in the SDP group were compared with the LDP group eyes. Moreover, a significant positive correlation was detected between the preoperative pupil size and contrast sensitivity or low contrast VA. No differences, however, were observed in distance and near VA as well as defocus curve between the SDP group eyes and the LDP group eyes.

Hütz *et al*.^8^ reported that reading ability becomes worse with low illumination in patients wearing a diffractive IOL, similar to our results. In other words, an additional decrease in contrast may be induced in small-pupil eyes by the lack of illumination in addition to the intrinsic low contrast of the diffractive IOL.

In general, the indications for MIOL must be more carefully assessed than those for conventional monofocal IOLs. Fujimoto *et al*.^9^ and Kawamorita *et al*.^10^ reported that preoperative pupil size is an important indication, and that a photopic pupil size greater than 3.0 mm is recommended for MIOL implantation. Moreover, Karhanová *et al*.^11^ reported that visual symptoms deteriorate significantly with temporal decentration of the MIOL in eyes with a large angle kappa. Thus, not only pupil size but also the positional relationship between the pupil center and the IOL center might be critical factors.

Artigas *et al*.^5^ reported that when using diffractive MIOLs, the optical image deteriorates with a small pupil, and there is an intrinsic optical loss with this type of IOL, using optical bench measurements with an eye model. To date, the study by Alfonso *et al*.^6^ of apodized diffractive-refractive MIOLs is the only clinical report describing the relationship between pupil size and visual function in eyes with a diffractive MIOL. In that study, the authors reported that eyes with large pupils registered better distance VA and distance contrast sensitivity and worse near VA than eyes with small pupils due to the energy (light) distribution produced by the apodized diffractive-refractive mechanisms. To the best of our knowledge, however, there are no published studies describing the clinical outcome of eyes with a small pupil and a full optic diffractive MIOL. On the other hand, in the present study of full optic diffractive IOLs, small pupil eyes had worse contrast than larger pupils, and postoperative pupil size positively correlated with contrast.

García-Domene *et al*.^12^ reported that the modulation in near visual acuity mildly increased with the pupil size through a full optic diffractive MIOL using an optical bench. And also in another experimental report using an apodized diffractive MIOL mounted optical bench system, the energy of the image increased with a pupil diameter up to 3.6 mm^13^, indicating that the same results are obtained even with an apodized diffractive MIOL when observed inside the diffractive zone. Our clinical results are in agreement with those two sets of model eye data.

The findings of this present study illustrate that preoperative pupil size should be carefully considered even for diffractive MIOL implantation due to the additional deterioration of contrast. Moreover, intraoperative multiple small sphincterotomies may be a useful surgical option to address such cases.

This study has some limitation. First, because deterioration of contrast in the diffractive MIOLs was not originally observed in eyes with a postoperative pupil size less than 3.0 mm, postoperative pupil size seems to be key. Koch *et al*.^14^ reported that preoperative pupil size does not always reflect postoperative pupil size. In our study, however, postoperative pupil size showed significant positive correlation in LDP group, while no significance was observed in SDP group that multiple small sphincterotomies were performed in some cases. Also it should be concerned that change of anterior chamber depth associated with cataract IOL surgery may affect the measurement of pupil diameter. So that in some cases, the surgeon cannot determine prior to surgery whether the MIOL should be implanted. And the second, the difference in the number of cases between the SDP and LDP groups may have been large. Power analysis indicated, however, that our study had sufficient statistical power for all of the main outcomes.

In conclusion, our findings revealed that postoperative pupil size positively correlates with contrast in diffractive IOL-implanted eyes. Eyes with a preoperative pupil size of 3.0 mm or less had worse contrast sensitivity than those with a preoperative pupil size greater than 3.0 mm, but when small pupils became larger than 3.0 mm postoperatively, the contrast sensitivity improved and was no longer significantly different from that of the larger pupil size group. Thus, although measurement of pupil size is a critical examination when considering the use of a diffractive MIOL, further studies are needed.
